# A Protocol for the Biomechanical Evaluation of the Types of Setting Motions in Volleyball Based on Kinematics and Muscle Synergies

**DOI:** 10.3390/mps9010006

**Published:** 2026-01-03

**Authors:** Valentina Lanzani, Cristina Brambilla, Nicol Moscatelli, Alessandro Scano

**Affiliations:** Advanced Methods for Biomedical Signal and Image Processing Laboratory, Italian Council of National Research (CNR), Institute of Intelligent Industrial Systems and Technologies for Advanced Manufacturing (STIIMA), 20133 Milan, Italy; cristina.brambilla@cnr.it (C.B.); nicolmoscatelli@cnr.it (N.M.); alessandro.scano@cnr.it (A.S.)

**Keywords:** biomechanical evaluation, kinematics, muscle synergies, setting motion, volleyball

## Abstract

Setting is a fundamental movement in volleyball. While there are several optimal interpreters of the role in professional play, there is a surprising lack of advanced measurement techniques for the evaluation of the movement from a biomechanical perspective. We proposed a comprehensive motion analysis protocol based on kinematics and motor coordination assessment (muscle synergies) for an in-depth analysis of the setting gesture. We also quantified the test–retest performance and discussed in detail the potential of the method. A single experienced player (age 27) tested and retested the protocol. The protocol was quite rapid to perform (about 30 min, including placement of kinematic and electromyography sensors on the patient’s body); we found high test and re-test consistency in different sessions within this participant (ICC > 0.90). These preliminary results suggest that the protocol could support the use of the state-of-the-art methods for motion analysis and biomechanics in volleyball and sports in general.

## 1. Introduction

Volleyball is one of the most popular Olympic sports, played between two teams of six players on a court divided by a net [1]. The game consists of a sequence of actions structured around strategic offensive and defensive plans. Specifically, volleyball is organized into attack and defense phases and is based on four fundamental skills: bump, spike, set and serve [2]. The set is the most important skill that enables the transition from defense to attack. Indeed, the setting motion represents the second contact with the ball in each action and allows the team to organize an effective offensive play and ultimately score a point [2]. Setting is the fundamental volleyball-specific skill executed by a specialized player known as the setter. The setter is responsible for organizing the offensive play, making the majority of tactical decisions and placing attackers in the optimal position to score points, which is crucial for securing the team’s success. Indeed, a large proportion of attacking efficiency depends on the quality of the set, and a strong relationship may exist between the setting performance and the final outcome of a match [3].

Despite the crucial role of setting in volleyball, relatively few studies have evaluated and monitored setter performance. In particular, only Cabarkapa et al. [2] assessed kinetic and kinematic characteristics of setting motions in volleyball players. Other studies assessed mainly the setting efficacy [3] or decision-making on the setting direction [4]. Most previous research has instead focused on other volleyball skills. In fact, in the scientific literature, the skills most frequently investigated are the spike, the serve, and the landing phase following a block jump [5,6,7]. Research in this area can generally be divided into kinematic analysis and EMG analysis, which are typically conducted independently of one another [8,9].

EMG analysis has primarily focused on examining the patterns and intensity of muscle activity during the spike and serve. These investigations aim to develop specific training programs and rehabilitation protocols designed to minimize the risk of injury [9]. EMG analyses are also frequently employed to explore muscle activation during jumping, with the objective of assessing its effects on joint mechanics, particularly in individuals with joint instability [10]. Additionally, several studies have investigated how muscle fatigue affects jump performance and execution [7].

Conversely, kinematic studies have predominantly concentrated on the analysis of the serve and spike, both to describe their general kinetic and kinematic characteristics [8,11,12,13] and to identify kinematic differences among various types of serves (standing serve, jump float serve, and jump spin serve) or between different volleyball-spiking jump techniques (hop-jump and step-close jump) [14,15]. Further research has focused on upper-limb kinematics, particularly on the shoulder joint, which plays a key role during these actions. Such studies aim to enhance understanding of injury prevention strategies and the effect of overuse on the shoulder complex [5]. Additional kinematic investigations have examined lower-limb kinematics during the landing phase following a block or spike jump, in order to evaluate the biomechanical impacts these skills exert on the knee and hip joints [1,16].

Overall, the existing literature demonstrates a greater prevalence of kinematic studies compared with EMG analyses. To the best of our knowledge, no studies to date have employed EMG-based muscle synergy extraction to investigate motor control strategies across different volleyball technical skills.

Among all volleyball fundamentals, the set remains the least studied skill, both from a kinematic and EMG perspective, likely because it is a highly complex and technically demanding movement. The few existing studies have mainly focused on kinematic, kinetic, and biomechanical aspects of setting motion, or have examined muscle activity during the execution of the set [2,3,17,18]. However, the setting is characterized by refined motor coordination to achieve an accurate and effective pass to the attacker, and therefore warrants further investigation on kinematics and neuromuscular control. Therefore, setting requires a dedicated assessment protocol because it involves distinct kinematic and neuromuscular characteristics due to the fine motor control of the forearm, highly precise movements, accurate timing, and different activation patterns with respect to spiking or serving.

Consequently, the present study proposes a novel comprehensive protocol for the analysis and evaluation of setting motion. In addition to examining the kinematics of upper limb movements, this protocol also investigates muscle synergies, a popular method for assessing motor coordination at the muscular level, to better understand how motor control is achieved during the setting motion. This combined approach aims to provide a deeper insight into how muscles coordinate to perform the set, thereby offering added value to the kinematic information that has already been analyzed in previous studies. Recent research has highlighted how instrumented biomechanical approaches can improve sport performance and support both training optimization and athlete assessment [19,20,21].

Finally, this study aims to develop a synthetic biomechanical assessment protocol integrating kinematic and electromyographical analyses to evaluate joint behavior and neuromuscular characteristics during movement. This protocol could be used by coaches to train setters and refine their setting technique. By analyzing differences among various types of sets, coaches can design more targeted training programs to optimize each setting action, using the identified biomechanical parameters (shoulder, elbow, wrist joint angles, as well as muscle synergies) as reference standards and providing instrumental feedback for technical improvement.

## 2. Experimental Design

### 2.1. Equipment and Sensors Positioning

**Figure 1:**
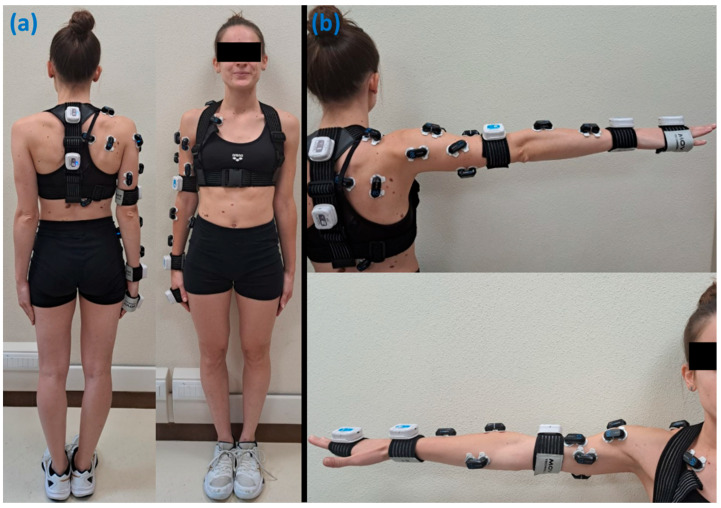
(**a**) Subject’s equipment to perform the test with the wearable inertial sensors and EMG electrodes placed on the right arm. (**b**) Each EMG electrode was placed on a specific muscle indicated in the list. 01: Latissimus Dorsi; 02: Lower Trapezius; 03: Middle Trapezius; 04: Upper Trapezius; 05: Infraspinatus; 06: Anterior Deltoid; 07: Middle Deltoid; 08: Posterior Deltoid; 09: Pectoralis Major; 10: Triceps Long Head; 11: Triceps Lateral Head; 12: Biceps Long Head; 13: Biceps Short Head; 14: Brachioradialis; 15: Flexor Carpi Radialis; 16: Extensor Carpi Radialis.

During the execution of setting actions, 5 small wearable inertial devices, Movit System G1 by Captiks, (Captiks S.r.l., Rome, Italy), and 16 wireless electromyographic (EMG) electrodes (Cometa S.r.l, Milan, Italy) were used to collect kinematic data and muscle electrical activations, respectively. Electromyographic data were obtained from 16 surface electrodes placed on the right upper limb and trunk, while kinematic data were recorded by three inertial sensors positioned on the right arm and two on the back, as shown in Figure 1. During data acquisition, only the dominant limb (right) was considered, assuming symmetrical behavior between the limbs. This assumption is based on the fact that both arms work together during the setting action to control the ball and generate the correct trajectory, implying that the movement is largely coordinated and symmetric. Moreover, further exploitations of our protocol may verify this aspect.

The inertial sensors (48 × 39 × 18 mm, device mass 27 g) are equipped with a tri-axial accelerometer (±156 m/s^2^), a tri-axial gyroscope (±2000 deg/s) and a tri-axial magnetometer (±4800 µT), and they acquired data with a sampling rate of 100 Hz. Sensors required a two-step calibration process to map the correct orientation of the body joints and to allow the reconstruction of kinematics and anatomical angles. The first step involved placing the inertial sensors on a calibration base and acquiring 3 orthogonal positions through two 90° rotational movements to define a unique reference coordinate system. Afterwards, the sensors were attached to the body at specific positions using elastic bands, and the second calibration step was performed. This step consisted of the acquisition of the T pose (standing position with arms parallel to the ground) to align the Movit System with the body coordinate system. Once calibration was completed, the Motion Studio system (Captiks S.r.l., Rome, Italy), was ready to collect data, and the player could begin performing setting movements.

To prevent drift and ensure the system remained properly calibrated, the second calibration step was performed after about 10 min of data acquisition. During this recalibration, the subject returned to the T-pose position, and the “Reposition” button in the Motion Studio software (3D edition v.2.3.47) was clicked.

The EMG electrodes recorded the activity of 16 muscles of the dominant upper limb and trunk: Latissimus Dorsi (LD), Lower Trapezius (LT), Middle Trapezius (MT), Upper Trapezius (UT), Infraspinatus (In), Anterior Deltoid (AD), Middle Deltoid (MD), Posterior Deltoid (PD), Pectoralis Major (PM), Triceps Long Head (TLongH), Triceps Lateral Head (TLatH), Biceps Long Head (BLongH), Biceps Short Head (BShH), Brachioradialis (Br), Flexor Carpi Radialis (FlexCR) and Extensor Carpi Radialis (ExCR), positioned according to the SENIAM guidelines when available for those muscles, and on the position at the middle of each muscle belly when guidelines were not available for specific muscles [22]. The skin surface was prepared by depilating and cleaning with alcohol to reduce skin impedance. Electrodes were circular (radius 8 mm), and the center-to-center electrode distance was 24 mm. EMG data were acquired with a sampling frequency of 2000 Hz.

### 2.2. Data Acquisition

The collected kinematic and EMG data were hardware synchronized through an analog trigger provided from the EMG to the Captiks system through a BNC cable. The data were visualized and stored in the EMG tools software (Version 8. Cometa, Milan, Italy) and in the Captiks Visualizer (Motion Analyzer).

## 3. Procedure

### 3.1. Experimental Protocol

**Figure 2:**
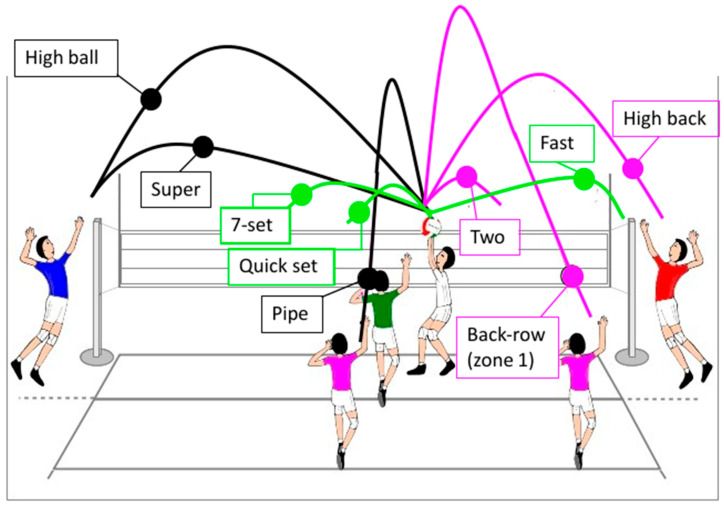
Types of setting simulated inside the laboratory. Black lines represent the ball set to the outside hitter, the green line represents the ball set to the middle blocker, and the pink lines are the ball set to the opposite hitter.

The movements of different types of volleyball setting were simulated in the laboratory by an experienced, healthy volleyball player (female, age 27 years, height 1.60 m, body mass 50 kg), with no neurological or orthopedic issues, with the feet on the ground (no jumps were performed). Specifically, simulating an optimal posture for the setter, at midway between positions 2 and 3 on the field, the simulation involved performing the arm and leg movements without a ball to execute nine type of setting actions: three directed to the outside hitter (a high ball, ‘super’ ball and pipe), three to the middle blocker (a quick set, a 7-set, and a fast), and three to the opposite hitter (a ‘two’, a high back set and a back-row set to zone 1) (Figure 2). Each type of volleyball setting was repeated 10 times in a randomized order, with a 2 s rest between repetitions, and setting motion was required to be completed with the hand remaining overhead for 2 s. After each set, the arms were put back, leaning along the body, simulating a resting state position for 1 min. The participant gave his informed consent; ethical approval was obtained from the CNR Ethics (Ethical Committee), and the experiment was conducted in accordance with the declaration of Helsinki.

### 3.2. Kinematics

The kinematic variables analyzed in this study included the time series of three shoulder joint movements: flexion-extension, abduction-adduction, and internal-external rotation; two elbow joint movements: flexion-extension and pronation-supination; and the wrist flexion-extension. These joint angles were assessed across all types of setting movements, and for each joint and movement type, the variability of the range of motion was then calculated. The segmentation of movement events was based on the flexion-extension angle of the elbow. For this specific aim, this signal was low-pass filtered with a 3 Hz cut-off frequency, and the resulting waveform was used to determine the onset and offset of each movement using a velocity profile algorithm. The onset and offset of each setting movement were extracted using an automatic segmentation pipeline based on a combination of derivative analysis and thresholding. First, for each setting motion, the first derivative of the filtered elbow flexion-extension signal was computed. Subsequently, a threshold was defined as a percentage of the derivative amplitude, ranging from 20% to 30% depending on the type of setting considered. These parameters were used to identify the main flexion peaks. Zero-crossings of the derivative were then detected to delineate intervals of slope inversion. Local minima were identified using a MATLAB 2023 (Mathworks, Natick, Massachusetts, United States)-based pipeline designed to detect only the negative peaks falling below a threshold defined as a percentage of the derivative amplitude. As for the detection of the maximum peaks, this percentage was adopted for the specific type of setting movement, varying between 20% and 30% of the derivative amplitude to account for differences in movement characteristics. Movement onset was assigned to the first zero-crossing preceding each major derivative peak, and movement offset to the first zero-crossing following each relevant local minimum.

Kinematic data of all joints considered, used for visualization and analysis, were instead low-pass filtered at 3 Hz. This processing step allowed for a clear view of how the different joint angles changed over time, removing motion artifacts.

To characterize the average joint-angle profiles for the different set types, the following processing steps were applied. For each set type, the repetition with the longest duration was first identified. All other repetitions of the same type were then resampled to match this duration. This approach allowed us to compute an average kinematic profile for each set type while preserving the natural differences in duration across the various types of sets.

### 3.3. EMG Pre-Processing and Muscle Synergies Extraction

In this study, to gain a better understanding of motor control during different volleyball setting movements, the spatial model of muscle synergy extraction was applied.

Before extracting muscle synergies from the EMG signals, some pre-processing steps were required to obtain a single EMG matrix containing all repetitions of the nine setting types performed by the subject. EMG data were high-pass filtered with a cut-off frequency of 10 Hz, full-wave rectified and low-pass filtered with a cut-off frequency of 5 Hz (Butterworth filter, 5th order). Movements were then aligned by considering the EMG signals starting 0.25 s before the task onset and ending 0.25 s after the offset, to ensure capturing the complete EMG waveform [23]. Successively, since the different sets had varying durations, all EMG signals were resampled to 100 samples from onset to offset, so that all repetitions could be concatenated for each setting motion. Signals were then normalized to the maximum value of the EMG envelope for each muscle across all trials [24,25,26]. This procedure resulted in one EMG matrix with M rows and K × T × N columns:

M was the number of muscles, K was the number of setting motions, T was the number of samples of each setting, and N was the number of repetitions.

The synergies extraction model decomposes the EMG signals into the product of time-invariant synergy vectors and time-varying activation coefficients [27]. Specifically, the concatenated EMG matrix was decomposed using non-negative matrix factorization (NMF) as $\sum_{i=1}^{S}c_{i}^{k,n}\left(t\right)w_{i}(m)$, where $w_{i}$ were the time-invariant (or spatial) synergy vectors representing the contribution of each muscle to each synergy, and $c_{i}$ the time-varying scalar activation coefficients for each synergy (*i* = 1, …, S), describing the temporal activation of each synergy during task execution [28]. To achieve unit vector synergies, each spatial synergy was normalized by the Euclidean norm of that synergy and the temporal coefficients were normalized by the reciprocal of the norm. The goodness of reconstruction was evaluated with an R^2^ value defined as $1-\frac{SSE}{SST}\;$ where SSE was the sum of the squared residuals and SST was the sum of the squared differences with the mean EMG vector [29]. Synergies were extracted from order 1 to order 16 (number of muscles), and the algorithm was applied 20 times, starting from different random initial conditions in order to avoid local minima. The number of extracted synergies was selected as the minimum number necessary to achieve an R^2^ value of at least 0.80.

### 3.4. Test–Retest

The protocol was repeated one week later. The same acquisition procedure was applied, ensuring that the simulated setting movements were performed under conditions comparable to those of the initial trial. The Captiks and EMG sensors were positioned on the same anatomical landmarks by the same operator in order to eliminate inter-operator variability and minimize variability across sessions. As in the first acquisition, both joint angle patterns and muscle synergies were analyzed.

To evaluate movement and protocol consistency between sessions, the similarity of muscle synergies and the correlation of the kinematic joint angle trajectories were examined. The similarity of the spatial structure of muscle synergies was quantified using cosine similarity, whereas the consistency of the temporal synergies’ patterns was assessed through the Pearson correlation coefficient (r). The repeatability of kinematic joint angle trajectories was, instead, evaluated using the Pearson correlation coefficient and the Intraclass Correlation Coefficient (ICC). The Pearson coefficient allowed quantification of trajectory similarity, while ICC provided a measure of consistency. Specifically, ICC (3, k) was adopted, as the analysis involved a single subject evaluated in two fixed sessions, and the mean of repeated trials was considered. This model was chosen because ICC is regarded as the standard coefficient for consistency assessment, since it accounts for both correlation and agreement between measurements [30].

## 4. Results

### 4.1. Joint Angles

The joint angles of the shoulder, elbow and wrist were calculated for each setting motion, including those directed to the outside hitter (high ball, ‘super’ ball and pipe), to the middle blocker (quick set, 7-set, and fast), and to the opposite hitter (‘two’ set, high back set and back-row set to zone 1). Since ten repetitions were performed for each type of set, the mean joint angle was computed for each joint, and the standard deviation was evaluated to assess the variability across repetitions during the execution.

**Figure 3:**
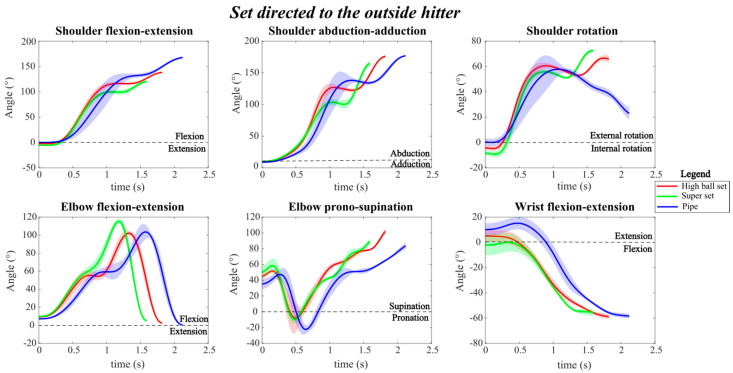
Joint angle profile during the three different types of sets directed to the outside hitter, with the corresponding standard deviations. The red line represents the high ball set, the green line represents the ‘super’ set and the blue line represents the pipe set.

Regarding the setting motions directed to the outside hitter (Figure 3), it was observed that the execution of the pipe set (to zone 6) required a longer duration compared to both the high ball and the super sets, as indicated by the more extended time course of the joint angle patterns. Furthermore, during the pipe set, the shoulder showed a greater flexion-extension at the end of the movement compared to the super and the high ball sets, reaching a flexion angle of approximately 150°.

The high ball and super sets exhibited very similar joint angle trends, with only minimal differences in the angular values achieved throughout the movement phases.

The elbow flexion-extension angle best highlighted the different movement speeds among the three sets: the peak flexion occurred at different times, with a higher and earlier peak (around 100°) in the super set, and a slightly lower and delayed peak in the pipe set. The time to peak elbow flexion was approximately 0.5 s earlier in the super set compared to the high ball set, and nearly 1 s earlier than in the pipe set.

The variability of the joint angle patterns, represented by the shaded areas, was slightly higher in the pipe set, particularly for shoulder and elbow movements, indicating greater dispersion in the angular trajectories across the repetition performed during the trial.

**Figure 4:**
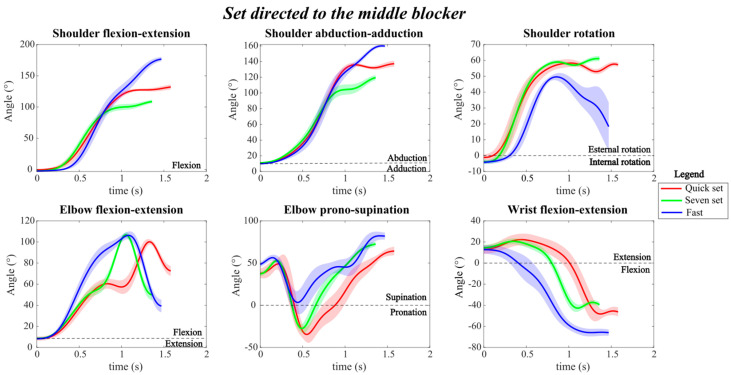
Joint angle profile during the three different types of sets directed to the middle blocker, with the corresponding standard deviations. The red line represents the quick set, the green line represents the seven set, and the blue line represents the fast set.

For the setting motions directed to the middle blocker (Figure 4), similar joint angle patterns were observed for shoulder and wrist flexion-extension, as well as for shoulder abduction-adduction and elbow pronation-supination. The main difference among the three sets was found in the final angular values. Indeed, the fast set reached greater shoulder extension and abduction angles (approximately 180° and 160°, respectively). While the seven set displayed much smaller amplitudes, with about 100° of shoulder extension and 110° of abduction. The quick set showed intermediate values between the two.

Although all three sets reached a similar final elbow pronation-supination angle, the seven and quick sets exhibited a larger range of motion throughout the setting movement. The fast set required a greater and more abrupt wrist flexion, reaching approximately 60° of flexion.

Observing the quick set, the arms were first raised above the head with the elbows flexed at approximately 60°. During the setting motion, the elbows were further flexed to about 100°, then extended slightly to propel the ball, finishing the movement with the elbows still flexed at around 70°. In contrast, during the fast and seven sets, the arms are brought directly above the head, and the elbows are flexed to approximately 100° to load the setting action. The movement was then concluded with the elbows in a more extended position, reaching about 40° of flexion.

Conversely, for shoulder rotation, the fast set showed a greater variation during the movement, reaching a peak of about 50°, which then decreased to approximately 20° by the end of the motion. In contrast, in both the seven and quick sets, once the peak rotation angle was reached, it remained relatively constant until the completion of the set.

Finally, the variability, represented by the shaded regions, appeared more pronounced in the fast set, particularly for shoulder and wrist movements, reflecting greater dispersion among repetitions performed during the task.

**Figure 5:**
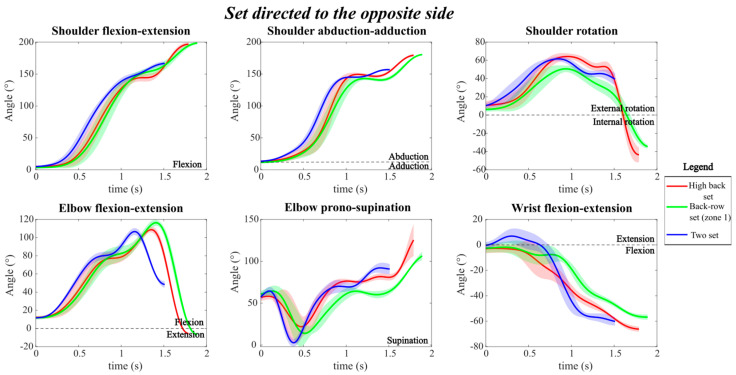
Joint angle profile during the three different types of sets directed to the opposite hitter, with the corresponding standard deviations. The red line represents the high back set, the green line represents the back-row set (zone 1) and the blue line represents the two set.

Figure 5 shows the setting motions directed to the opposite hitter.

All joint angles exhibited a similar overall profile across the three types of sets directed to the opposite hitter, with some variations in the final angular values reached at the end of the movement. Specifically, both the high back set and the back-row set from zone 1 reached comparable final joint angles across all joints, whereas the two-set showed distinct final values, except for wrist flexion, which was similar among all three sets (approximately 60° of flexion).

At the end of the movement, the two-set displayed smaller shoulder extension and abduction angles (approximately 130° and 120°, respectively) and a reduced elbow pronation-supination angle compared to the other two opposite sets. In addition, in the two-set, the elbows remained flexed (around 50°) at the end of the motion, while in the high back and back-row sets, the elbows were extended at the end of the movement.

Regarding shoulder rotation, a greater range of motion was observed for the high back set and the back-row set from zone 1, with the angle varying from 0° to −40° after reaching a peak of approximately 60° of rotation. In contrast, in the two-set, shoulder rotation remained constant after reaching a similar peak value.

For wrist flexion-extension, all three sets exhibited comparable temporal patterns and reached the same final angle; however, during the execution of the two-set, wrist flexion occurred more rapidly, as shown by the steeper slope of the curve.

Moreover, the two-set required a faster overall execution, as indicated by the shorter duration of the joint angle trajectories across all degrees of freedom. The elbow flexion-extension angle best highlighted this difference: the peak flexion occurred approximately 0.5 s earlier in the two-set than in the high back and back-row sets, which exhibited slower movements characterized by wider flexion-extension excursions.

Finally, the variability, represented by the shaded areas, appeared slightly higher in the high back set, particularly for shoulder rotation and elbow pronation-supination, indicating greater dispersion among repetitions compared to the two and back-row sets.

### 4.2. Range of Motion

**Figure 6:**
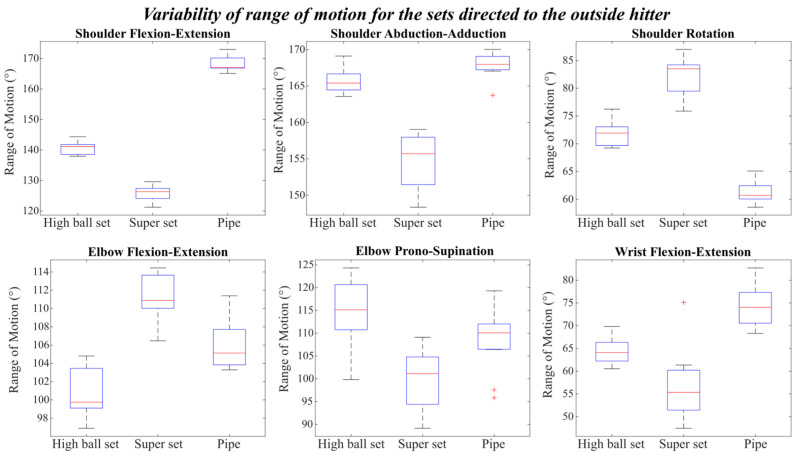
Variability of the Range of motion for the set directed to the outside hitter.

For each type of setting, this protocol analyses the range of motion of all joints and their variability across the repetition. As illustrated in Figure 6, referring to the sets directed to the outside hitter, all joint movements exhibited notable differences in the range of motion among the three types of setting: high ball, ‘super’ ball, and pipe.

Overall, the super set displayed a larger inter-trial variability, as reflected by the wider boxes in the plots, compared to both the high ball and pipe sets. The only exceptions were shoulder flexion-extension and elbow flexion-extension, where the three conditions showed comparable dispersion around the median values.

Regarding the absolute range of motion, the pipe set was characterized by the largest shoulder excursion in flexion-extension and abduction-adduction, reaching values close to 170°. The wrist flexion-extension also exhibited a wider amplitude in the pipe set, with a median of around 75°. Conversely, the shoulder rotation range was smaller during the pipe set (median ≈ 60°) compared to the high ball (≈75°) and especially the super set (≈85°).

The super set generally shows a smaller range of motion in most degrees of freedom, except for elbow flexion-extension, where it reached the highest values (up to 110°), and for shoulder rotation, which also showed relatively high excursions. The high ball set, on the other hand, produced the greatest motion amplitude in elbow pronation-supination, with a median of around 115°.

Among all joints, shoulder flexion-extension presented the lowest variability across all three setting types, indicating a more consistent motion pattern among repetitions.

**Figure 7:**
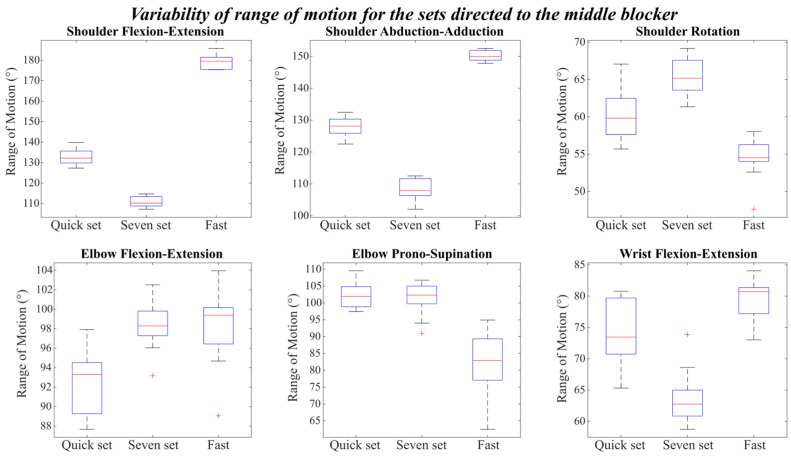
Variability of the Range of motion for the set directed to the middle blocker.

Similarly, the three types of setting motion directed to the middle blocker showed distinct differences in both the magnitude and variability of joint range of motion (Figure 7).

In general, the seven and the fast sets exhibited opposite trends across several joints, with an inverse relationship between their range of motion values; when one showed higher amplitudes, the other showed smaller ones. Specifically, during the fast set, the shoulder reached the highest amplitudes in both flexion-extension and abduction-adduction, with median values around 180° and 150°, respectively. These movements were characterized by low variability across repetitions, as indicated by the narrow boxes. In contrast, during the seven sets, the shoulder exhibited smaller ranges, approximately 110° in both flexion and adduction, with a similarly limited variability.

A comparable pattern was observed for the wrist flexion-extension, where the fast set showed a larger excursion (around 80°) than the seven set (around 60°). However, unlike shoulder movements, wrist flexion-extension suggested greater variability, particularly in the fast set, where the values ranged between 75° and 83°.

Regarding shoulder rotation, the seven set presented the largest range of motion, followed by the quick set, while the fast set showed the smallest rotation (approximately 55°).

For elbow flexion-extension, the quick set showed the lowest values, whereas the seven and fast sets reached similar and higher ranges, with a median value close to 100–102°. The quick set also displayed the widest inter-trial variability for this joint, as evidenced by the taller boxes and extended whiskers.

In the case of elbow pronation-supination, the quick set and seven set showed comparable and relatively high median values, while the fast set exhibited smaller and more variable ranges, fluctuating approximately between 75° and 90°.

Overall, across all conditions, the shoulder flexion-extension and abduction-adduction movements were those showing the most consistent behavior across repetitions, whereas wrist and elbow motion tended to exhibit higher inter-trial variability, particularly during the quick and fast sets.

**Figure 8:**
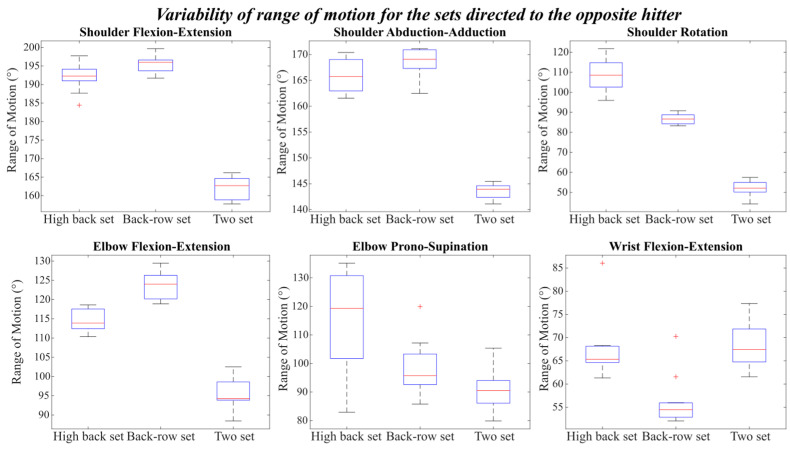
Variability of Range of motion for the set directed to the opposite hitter.

Finally, Figure 8 shows the variability of the joint range of motion for the setting motions directed to the opposite hitter: high back, back-row, and two-set.

As observed for the previous setting types, all joints showed distinct differences in both magnitude and variability of range of motion across the three conditions.

In particular, the figure displayed that the back-row set required the greatest joint excursions, especially for the shoulder and elbow flexion-extension movements. Specifically, the shoulder flexion-extension reached values up to approximately 198° in the back-row set, compared with about 192° in the high back set and 164° in the two-set. A similar pattern was observed in elbow flexion-extension, where the back-row set displayed the highest range of motion (around 125°), followed by the high back set (≈115°) and the two-set (≈95°).

The shoulder abduction-adduction also reached its maximum amplitude during the back-row set, with peaks of about 170°, whereas both front-row sets (high back and two-set) showed smaller ranges, between 145° and 165°.

Regarding shoulder rotation, the high back set showed the largest angular displacement, reaching around 110°, followed by the back-row set (≈85°) and the two-set (≈50°).

For the elbow pronation-supination, the high back set exhibited the widest inter-trial variability, as indicated by the taller boxes and longer whiskers, with values ranging from 100° to 130°. The back-row set and two-set, on the other hand, showed lower and more consistent ranges (around 110° and 95°, respectively).

In the case of wrist flexion-extension, the back-row set displayed the lowest range of motion and smallest variability, with values between 50° and 55°. The two-set and high back set both showed higher and comparable ranges, around 65°, although the two-set presented a greater inter-trial variability, with values spanning from 60° to 75°.

Overall, the two sets consistently showed the lowest joint ranges of motion across all degrees of freedom, together with a moderate-to-low variability, except for the wrist flexion-extension, where the variability across repetitions was markedly higher.

### 4.3. Muscle Synergies

**Figure 9:**
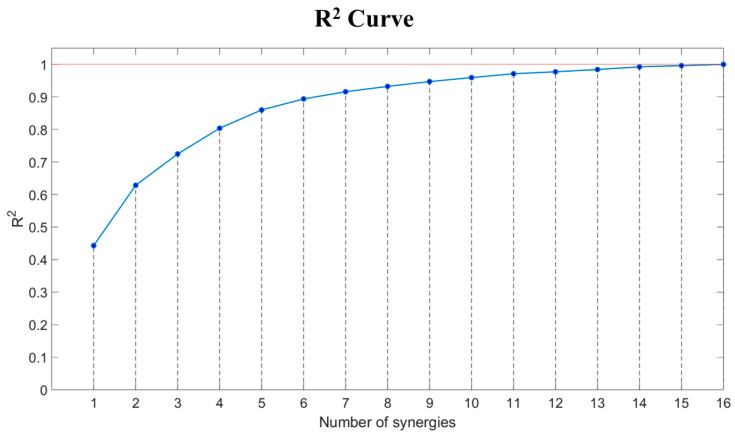
Reconstruction R^2^ curve for muscle synergies.

In this study, five distinct muscle synergies were identified to characterize the patterns of muscle activation contributing to the performance of different types of setting motions. Five synergies were selected because this is the minimum number required to reach the established threshold of 0.80 (R^2^ > 0.80) (Figure 9).

**Figure 10:**
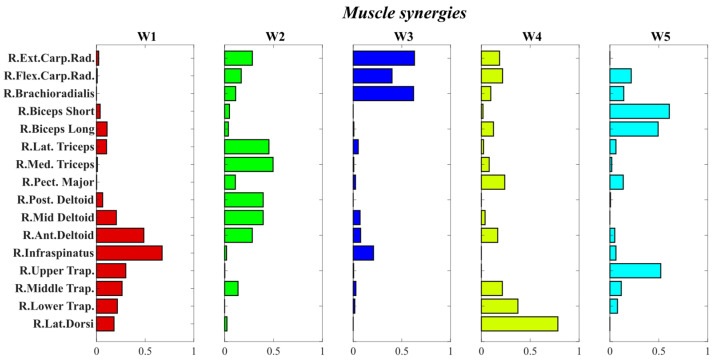
Five distinct muscle synergies, which identify the patterns of muscle activation contributing to the performance of different types of sets. Within each synergy, the bars quantify the relative activation contribution of each muscle, indicating its weighting within the synergy vector.

Figure 10 illustrates the spatial components of these synergies, represented by specific coefficients that describe the coordinated activation of multiple muscles. Synergy W1 was characterized by a strong activation of the Middle and Lower Trapezius, Infraspinatus, Anterior Deltoid, Middle Deltoid, and Biceps Long Head. Synergy W2 exhibited activation of the Long and Lateral Triceps, Anterior, Middle, and Posterior Deltoid, Infraspinatus, Middle Trapezius, and the wrist and forearm muscles (Flexor and Extensor Carpi-Radialis and Brachioradialis). Synergy W3 predominantly involved the forearm and wrist muscles, including Brachioradialis, Flexor Carpi-Radialis, and Extensor Carpi-Radialis. Synergy W4 displayed a pattern of activation primarily involving the Latissimus Dorsi and Lower Trapezius, and with reduced activity involving Pectoralis Major, Anterior Deltoid, Triceps, Biceps, and wrist muscles. Finally, synergy W5 showed a more diffuse muscle activation, with a notable contribution from Biceps Long and Short and Upper Trapezius, and with reduced activity of Flexor Carpi Radialis, Brachioradialis, Triceps, and Pectoralis.

**Figure 11:**
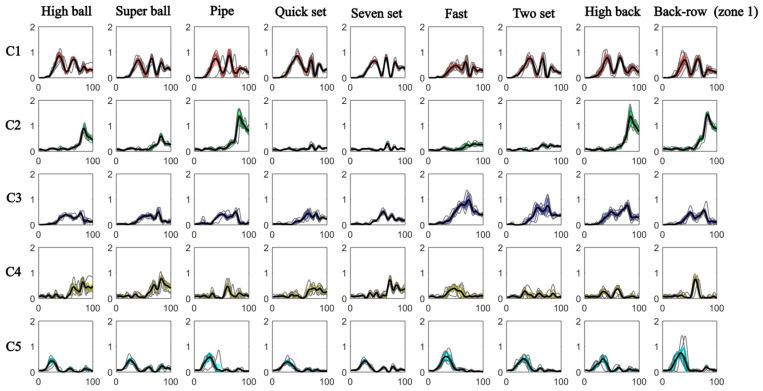
Temporal activation coefficients of muscle synergies.

Moreover, Figure 11 illustrates the temporal activation coefficients of muscle synergies, showing that synergy W5 was the first to be activated. Specifically, both the Long and Short heads of the Biceps Brachii, along with the forearm muscles, Brachioradialis and Flexor Carpi Radialis, were recruited to allow the subject to flex the elbow and wrist in order to initiate the arm lifting movement. Subsequently, the Medial, Anterior, and Posterior Deltoid, together with the Middle and Lower Trapezius, became engaged, enabling the subject to raise the arms above the head to perform the setting movement by flexing and extending the elbows (W1). Then, synergies W3 and W4 were activated almost simultaneously: W3 contributed to wrist movements that simulated ball handling (in and out), while W4 mainly involved back muscles, which played a stabilizing role during the pushing phase. Finally, synergy W2, characterized by the recruitment of the Long and Lateral Triceps together with the Medial and Anterior Deltoid, was activated at the end of the movement. These muscles were essential in the final phase to simulate the push of the ball toward the setting zone while keeping the arms extended overhead.

Interestingly, synergy W2 displayed task-dependent modulation. It was more strongly activated during the sets directed to the outside hitter, as well as during high back-row sets and high sets to the opposite hitter. Conversely, its activation was almost negligible, showing temporal coefficients close to zero, during quick sets to the opposite hitter (two-set) and during sets directed to the middle blocker.

### 4.4. Test–Retest

#### 4.4.1. Muscle Synergies Similarity

**Figure 12:**
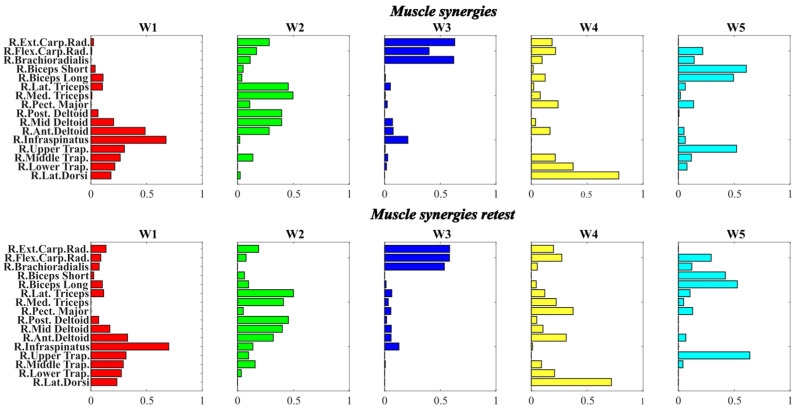
Comparing between muscle synergies across the two sessions.

When comparing the muscle synergies extracted from the two sessions (Figure 12), all exhibited a consistently high degree of similarity, with an average value of approximately 0.96. Table 1 shows the results of cosine similarity between synergies across the two sessions.

The results showed high similarity values across all synergies, ranging from 0.93 to 0.97, indicating that the structure of the synergy patterns remained highly consistent across the analyzed trials. Specifically, W1, W2, W3, and W5 showed similarity indices above 0.95, suggesting a very stable muscle weighting distribution. W4, which represented the synergies responsible for controlling the postural stability, exhibited a slightly lower similarity (0.93), indicating a somewhat greater variability in its structure, possibly reflecting condition-dependent adjustments in muscle coordination.

Moreover, assessing the correlation between the temporal coefficients of muscle synergies, the results are summarized in Table 2.

The results revealed high and significant correlations (*p* < 0.001) for most synergies across all setting types, indicating a quite strong temporal consistency of the synergy activations between sessions.

For the settings directed to the outside hitter (high ball, super ball, and pipe), the high ball set showed consistently high correlations across all synergies (r = 0.84–0.98), reflecting a stable activation timing. The super set also displayed strong correlations (r = 0.86–0.98), except for slightly lower values observed in C1 and C2. The pipe set exhibited greater variability, with very high correlations for C1, C2, and C5 (r > 0.90), but lower values for C3 (r = 0.73) and C4 (r = 0.74), suggesting more variable timing for those synergies between sessions.

For the middle blocker sets (quick, seven, and fast), the results showed more heterogeneous correlations. The quick set displayed high correlations for most components (r = 0.91–0.94) but lower values for C2 (r = 0.74) and C4 (r = 0.75). The seven set revealed a very low correlation for C2 (r = 0.36), whereas all other synergies remained highly consistent (r = 0.90–0.97). The fast set followed a similar trend, showing strong correlations for most synergies but a pronounced drop in C4 (r = 0.47).

For the opposite hitter settings (two, high back, and back-row sets), the correlations were again generally high. The two-set showed very strong similarities across most synergies (r = 0.94–0.98), except for a moderate correlation in C4 (r = 0.62). The high back and back-row sets both presented excellent correlations for C2 and C5 (r = 0.98–0.99), indicating a high reproducibility of these synergy timings across sessions, whereas C4 showed slightly lower values (r = 0.79–0.89).

In summary, these results preliminarily suggest that the temporal structure of the muscle synergies was generally preserved between the two sessions for all setting types. The only exceptions were C2 in the seven-set and C4 in the fast and two sets, which showed reduced temporal consistency, indicating a possible sensitivity of these specific synergies to contextual or executional variations between sessions.

#### 4.4.2. Pearson’s Coefficient and Interclass Correlation Coefficient for the Kinematic Patterns of the Joint Angles

For each type of set, the Pearson correlation coefficient (r) and the ICC were calculated by comparing the kinematic patterns of the analyzed joint angles across the two sessions. The results for sets directed to the outside hitter are summarized in Table 3, those for sets directed to the middle blocker are reported in Table 4, and those for sets directed to the opposite hitter are presented in Table 5.

The setting motions directed to the outside hitter showed a highly consistent kinematic pattern of joint angles between the two sessions. In fact, the high ICC values (ICC > 0.90) and Pearson’s correlation coefficients (r > 0.90) indicated excellent agreement between the corresponding curves, which exhibited very similar trends. The only joint angle showing a slight decrease in consistency was the elbow pronation-supination angle, which shifted from excellent consistency in the high ball and pipe set to good consistency in the super ball set, showing also a wider CI that indicated uncertainty.

All joint angles during the execution of quick and seven sets displayed very similar kinematic trends across the two sessions (ICC > 0.90), with strong linear correlations (r > 0.90). During the fast set, however, the pattern of joint angles became more variable, particularly for shoulder rotation and elbow pronation-supination, where the correlation dropped to around r = 0.86, and consistency decreased from excellent to good (0.75 < ICC < 0.90). Moreover, the CI was higher, reflecting more uncertainty in the measures.

The setting movements directed to the opposite hitter showed joint angles that were highly repeatable and strongly correlated, except for shoulder rotation and elbow pronation-supination. Shoulder rotation in all three sets exhibited a highly variable pattern across the two sessions, with moderate consistency (0.50 < ICC < 0.75). Specifically, during the high back set, shoulder rotation not only suggested a moderate repeatability but also a weak linear correlation between the curves obtained in the two sessions. Finally, the elbow pronation-supination angle showed moderate consistency (0.50 < ICC < 0.75), with Pearson’s correlation values ranging between 0.79 and 0.81. Also in this case, the CI was higher for both shoulder rotation and elbow pronation-supination, suggesting a lower consistency of these measures.

Overall, these results indicated that the kinematic patterns of the setting movements were highly consistent between sessions, with the exception of rotational components, particularly in shoulder rotation and elbow pronation-supination.

## 5. Discussion

### 5.1. Summary of Results and Added Value of the Protocol

This study proposed an experimental protocol designed to simultaneously measure kinematic parameters and muscle activity during the execution of a series of volleyball setting movements. Although apparently simple, the setting motion represents a highly refined motor task that requires great precision and coordination to be performed effectively, enabling the attacker to successfully complete the play.

The proposed protocol integrates synchronized measurement systems that allow the acquisition of a detailed kinematic profile of multiple joint angles, including shoulder, elbow, and wrist flexion-extension; shoulder abduction-adduction and rotation, and elbow pronation-supination, together with the muscle activation patterns of the main muscles involved.

By applying the developed protocol in two separate sessions using a test–retest procedure, we preliminarily suggested that both the muscle synergy patterns and the kinematic trajectories of the analyzed joint angles were consistent across sessions. These findings suggest that the proposed setup provides objective and repeatable measures, making it a valuable tool for the technical assessment of athletes’ performance and for monitoring changes in motor strategies across different training sessions or performance conditions. Furthermore, it would be possible to develop a comprehensive biomechanical map of the setting motion, offering valuable information for precise movement assessment and providing coaches with objective indicators to evaluate an athlete’s performance and technical proficiency.

### 5.2. Expected Outcomes and Application of the Protocol

This protocol would allow a technical evaluation of athletes by assessing both their initial performance level and improvements achieved through training. Specifically, it allows the identification of instrumental parameters, such as the joint angles of the elbow, shoulder, and wrist, that can serve as quantitative references to monitor changes in an athlete’s performance over time.

Moreover, by analyzing the EMG signals recorded during the acquisitions, it is possible to investigate how the muscles are engaged throughout the setting motion. This includes determining which muscles are activated and to what extent (spatial components of muscle synergies), as well as identifying the specific phases of the movement in which each muscle contributes (temporal modulation of muscle synergies).

The combined analysis of kinematic variables and muscle synergies makes it possible to identify coordination patterns and timing variation that are not easily detectable through visual observation alone [31]. This is particularly valuable for designing targeted training programs, allowing to refine specific phases of the setting motion, improving the smoothness and precision of the overall execution. Furthermore, this approach can be applied in rehabilitation contexts to determine the most effective strategies for restoring proper movement following an injury or for assessing the recovery level of an athlete after a rehabilitation or retraining period.

### 5.3. Validity and Methodological Limitations

The protocol developed in this study should be regarded as a preliminary assessment tool, and several limitations need to be taken into consideration.

First, only a single pilot participant was included in the study. While this allowed us to verify the feasibility and sensitivity of the protocol to be verified, the results cannot be generalized without testing a larger sample of athletes. Therefore, assessing the consistency of the protocol requires data from a greater number of subjects.

Additionally, the participant, although an experienced volleyball player competing in a regional division D championship, was not a professional setter. Her level of expertise may have influenced the execution of the movements and the resulting data. Therefore, if this protocol were applied using a test–retest procedure with a professional setter, the repeatability and consistency between the two sessions would likely be higher. Nevertheless, this protocol is designed to be applicable to both recreational and elite athletes.

Another limitation concerns the equipment used for kinematic measurements. The sensors were attached using elastic bands, which could slightly shift or slide during movement, potentially introducing small variations in the joint angle trajectories. Minor calibration drifts might also occur during motion execution, leading to slight differences in the measured range of motion. Therefore, a frequent recalibration of sensor placement is essential to minimize these variations.

During the retest phase, small differences in the placement of the bands and the EMG electrodes may have affected the repeatability of the recordings. To reduce this source of error, the same operator should place all sensors, minimizing inter-operator variability across sessions.

Furthermore, surface EMG measurements have intrinsic limitations and are influenced by variability related to signal pre-processing. Currently, there is still no universally accepted standard pipeline for EMG signal normalization and filtering in the literature, which may affect the interpretation of the extracted muscle synergies. Therefore, to enhance the generalizability of the results and allow meaningful comparisons across studies, it is necessary to define a standardized reference pipeline that will be consistently applied whenever this type of protocol is used.

Finally, the results of the test–retest analysis might have been influenced by the time interval between sessions. Learning effects, differences in the player’s daily condition, or slight inconsistencies in movement execution, particularly since the subject was not a professional athlete, could have affected performance stability.

In conclusion, while the stability and repeatability of the proposed protocol have been preliminarily suggested, its applicability should be further confirmed on a larger and more heterogeneous sample of participants.

### 5.4. Test–Retest Reliability

To assess the consistency of the protocol, both the ICC and the Pearson correlation coefficient were calculated on the kinematic profiles of the joint angles and on the temporal components of the muscle synergies. These two indices were selected because they respectively allow the evaluation of whether the shape of the curves is consistently repeatable over time (ICC) and whether the curves from the two sessions are linearly correlated (r).

Different types of ICC can be used depending on the study design and the purpose of the consistency assessment. In this study, the ICC (3, k) model was adopted because the same evaluator performed the measurements in both sessions, and the data were collected from a single participant who performed nine types of sets, each repeated ten times. The repetitions were then averaged to obtain one representative curve per session, allowing a comparison of the overall kinematic trends between sessions.

Regarding the kinematic profiles of the joint angles, the results showed a high level of reproducibility between the two sessions (ICC > 0.80; r > 0.78), with only minor variations observed in the rotational components, particularly in shoulder rotation and elbow pronation-supination angles. These angles also showed wider CI, reflecting the uncertainty of the measures. Unlike flexion-extension and abduction-adduction angles, which can be more easily controlled during the task, rotational angles are more difficult to standardize because they depend on fine rotational adjustments of the limbs. This observation aligns with findings reported in previous studies [32,33]. Consequently, this protocol may be better suited for analyzing movements that primarily involve joint flexion-extension, where rotational components are minimal or constant and have little impact on the overall movement, as is the case in the setting motion. Even small variations in arm elevation or wrist rotation during the simulated ball release can noticeably affect these angles [34]. Indeed, since the gesture was simulated rather than performed with an actual ball, the kinematic profiles might have been further influenced by differences in movement intensity and explosiveness, which can slightly alter the motion pattern [35,36].

Moreover, the sensitivity and calibration stability of the measurement system may also contribute to variability, potentially affecting the overall repeatability of the kinematic curves.

In contrast, the repeatability of the temporal coefficients of the muscle synergies was evaluated using only the Pearson correlation coefficient to determine whether the temporal patterns were consistent across sessions. The results indicated that the temporal structure of the synergies was well preserved between sessions for all types of sets, with the exception of seven, fast, and two sets. In these cases, variations were observed in the temporal structure of synergies W2 and W4, which are responsible for maintaining posture during the different phases of movement (W4) and for keeping the arms extended above the head after the ball release (W2). Synergy W4 primarily activated the back and shoulder muscles, with secondary involvement of other muscles, to stabilize the movement. This stabilizing function is reduced in these rapid setting motions, as body control and stabilization become more complex and less consistent. As a result, the temporal component of this synergy was less activated and occurred at different phases of the movement, making it more variable and less repeatable across sessions. Similarly, synergy W2 showed minimal temporal activation during the final phases of the movement in these rapid setting motions, since the arms remained above the head for only a brief period. This limited activation makes it difficult to obtain a consistent and repeatable activation pattern across sessions.

### 5.5. Interpretation of Muscle Synergies

The presence of 5 muscle synergies (R^2^ > 0.80) suggests that the setting motion is controlled through coordinated subsystems that enable adequate motor control for the execution of the task [37]. However, it should be noted that the structure of the synergies may reflect both biomechanical constraints and individual technical strategies. Therefore, a dual approach could be adopted, on one hand, to refine the technical execution of the movement, and on the other, to analyze the biomechanical and motor control aspects underlying the gesture, with the aim of achieving optimal performance.

A detailed analysis of the extracted synergies reveals that each one corresponds to a specific phase of setting motion. The first synergy to be activated (W5) primarily involves the Biceps Brachii and Upper Trapezius, which initiate the motion by slightly elevating the shoulders, flexing the elbows, and moving the arms forward. This is followed by synergy W1, characterized by the activation of the back muscles (Latissimus Dorsi, Upper, Middle, and Lower Trapezius and Infraspinatus) together with the shoulder muscles (Posterior, Middle, and Anterior Deltoid). These muscles are responsible for raising the arms overhead and maintaining their position during the elbows’ flexion-extension movements, which in turn involve activation of the Biceps Brachii and Lateral Triceps.

Subsequently, synergy W3 becomes active, engaging the forearm muscles involved in wrist flexion and extension (Brachioradialis, Flexor Carpi Radialis, and Extensor Carpi Radialis). This synergy represents the phase in which the ball contacts the hands and is then pushed upward toward the attacker. Shortly after, synergy W4 is recruited, mainly activating the Latissimus Dorsi and Lower Trapezius, which help stabilize the body during the ball-pushing phase. Finally, synergy W2 is activated, involving the Triceps, shoulder muscles, and the Flexor Carpi Radialis, which are essential in the final stage of the setting motion to keep the arms extended above the head and complete the ball release. Interestingly, this synergy exhibits task-dependent modulation. In back-row and pipe sets, W2 shows stronger activation at the end of the movement compared to front-row sets to the outside hitter and the high back set to the opposite hitter. In these back-row and pipe sets, the arms must be more extended to send the ball farther behind the net. In contrast, during high ball, super ball, and high back sets, W2 displays moderate activation, as the arms remain fully extended to complete the movement in the correct direction. Conversely, in the sets directed to the middle blocker and in the two-set to the opposite hitter, W2 activation is almost absent, since in these cases the arms do not reach full extension at the end of the motion. Instead, they remain slightly flexed, as these types of sets require greater wrist flexion-extension to ensure that the ball reaches the attacker quickly and at a lower trajectory.

Overall, the extracted synergies accurately describe the distinct phases of the volleyball setting motion. Therefore, analyzing these synergies in detail can provide valuable insights into whether the muscles are activated efficiently and with proper timing during execution, offering a powerful tool for evaluating and optimizing athletic performance [38].

### 5.6. Practical Applications for Coaches and Clinicians

The application of this protocol in volleyball-specific settings or within a rehabilitation studio could lead to valuable insights and practical benefits for both coaches and clinicians. For coaches, it can serve as a useful tool to assess the performance level of their athletes and to design targeted training programs aimed at improving specific technical aspects of performance. For physiotherapists, it could potentially assist in determining when an athlete is ready to safely return to training after an injury.

By analyzing joint angles, particularly those of the shoulder, elbow, and wrist, it would be possible to identify the optimal joint configurations that produce the desired ball trajectory for different types of sets. This information could help coaches refine technical execution, ensuring that athletes perform each type of set in the most efficient and effective way during gameplay.

Moreover, the study of muscle synergies could provide additional insights for both coaches and physiotherapists, especially in post-injury rehabilitation. Comparing synergy patterns between healthy athletes and those recovering from injury could reveal how muscle coordination changes after a trauma [38,39]. Such differences, whether in spatial components or in activation timing, may indicate compensatory mechanisms or altered motor control [40]. Consequently, physiotherapists and coaches could design specific exercises aimed at restoring balanced muscle activation patterns and re-establishing the athlete’s pre-injury motor control, thereby potentially facilitating a safe return to play. In particular, to determine an athlete’s readiness to resume sport, the physiotherapist should conduct a re-assessment of the athlete’s muscle synergies and movement kinematics following a period of targeted rehabilitation. These parameters should then be compared with those of healthy athletes to verify whether appropriate motor control and joint function have been restored. If so, the physiotherapist may authorize the athlete’s return to sport, while also recommending strengthening and maintenance exercises to reduce the risk of new injuries or recurrences.

Another relevant aspect is the assessment of how joint kinematics and muscle synergies change under fatigue conditions, such as during prolonged matches [41]. Fatigue may reduce the efficiency and precision of the movement. By analyzing performance parameters under these conditions, coaches could develop strategies to improve players’ endurance and maintain gesture effectiveness even under physical stress.

Finally, evaluating inter-limb symmetry during the setting motion could provide further valuable information. A proper set requires the ball to make simultaneous contact with both hands and to be pushed upward toward the attacker with perfectly synchronized arm movements. Therefore, ensuring symmetrical and well-balanced arm motion is crucial for technical accuracy. Coaches could apply this protocol to both the right and left upper limbs to obtain a detailed assessment of potential differences in joint angles or muscle activation patterns. This approach would allow them to identify any asymmetries with greater precision and correct them through targeted custom training programs to enhance bilateral coordination and movement symmetry.

### 5.7. Suggestions for Protocol Improvement

The present findings provide a solid methodological foundation for future investigations aimed at further validating and extending the proposed protocol.

The protocol validated in this study could be applied to a larger cohort to determine whether the findings observed in the pilot participant are generalizable to a broader population. Including athletes with different levels of expertise would also provide valuable insight into how both the correlations between the kinematic profiles of joint angles and the structure of muscle synergies vary across skill levels, from novice to elite players.

Future studies could also evaluate the setting motion under more game-like conditions, incorporating the presence of the ball to investigate whether, and to what extent, the simulated movement differs from the real execution. Further analyses could compare different execution contexts, such as static sets performed without jumping, jump sets, and sets executed during competitive gameplay versus isolated technical drills.

Moreover, since the setting motion is a complex task that requires proper coordination of the legs and trunk, a valuable extension of this work would be to include additional sensors on the lower limbs. This would allow for a more detailed analysis of knee flexion-extension angles and trunk inclination.

In addition, increasing the number of repetitions for each set type could further reduce measurement noise and improve the consistency of the collected data.

Defining reference parameters could be useful in helping coaches and practitioners design targeted training to refine technical execution. Moreover, the present methodological framework could be extended to other key volleyball skills, such as the bump, serve, or spike, thereby broadening its applicability.

Regarding EMG data analysis, future developments should consider implementing a cross-validation approach to objectively define the optimal number of synergies required to describe the movement. Establishing a standardized pre-processing and normalization pipeline would also be essential to ensure methodological consistency and comparability of results across laboratories.

In summary, several methodological improvements could enhance the robustness and versatility of this protocol, making it a reproducible and transferable tool for studying complex motor tasks across different volleyball techniques, experimental conditions, and athlete performance levels.

## 6. Conclusions and Future Perspective

The protocol developed in this study represents a first-step, preliminary objective procedure for assessing the setting motion in volleyball. Future research should expand its validation by including a larger sample of athletes and by further evaluating its clinical and performance relevance. An important step toward applying this protocol in real training environments is to first verify its validity in the laboratory by performing the setting movement with the ball. Once this aspect has been validated, it would be interesting to collaborate with volleyball practitioners, implementing the protocol in real training environments to assess athletes’ performance. This approach would allow us to evaluate the practical applicability of the protocol and help bridge the gap between laboratory-based analyses and on-court performance, ultimately promoting a more evidence-based approach to training, performance optimization, and injury prevention.

## Figures and Tables

**Table 1:** Similarity values comparing synergies extracted across the two sessions.

	W1	W2	W3	W4	W5
Similarity	0.97	0.96	0.97	0.93	0.96

**Table 2:** Pearson’s coefficient and *p*-value were evaluated by comparing the temporal coefficients of muscle synergies across the two sessions.

	*C*1	*C*2	*C*3	*C*4	*C*5
High ball	0.95 (<0.001)	0.98 (<0.001)	0.84 (<0.001)	0.95 (<0.001)	0.95 (<0.001)
Super ball	0.86 (<0.001)	0.91 (<0.001)	0.91 (<0.001)	0.98 (<0.001)	0.98 (<0.001)
Pipe	0.93 (<0.001)	0.99 (<0.001)	0.73 (<0.001)	0.74 (<0.001)	0.93 (<0.001)
Quick set	0.91 (<0.001)	0.74 (<0.001)	0.94 (<0.001)	0.75 (<0.001)	0.93 (<0.001)
Seven set	0.94 (<0.001)	0.36 (<0.001)	0.96 (<0.001)	0.90 (<0.001)	0.97 (<0.001)
Fast	0.84 (<0.001)	0.93 (<0.001)	0.90 (<0.001)	0.47 (<0.001)	0.92 (<0.001)
Two	0.98 (<0.001)	0.94 (<0.001)	0.98 (<0.001)	0.62 (<0.001)	0.98 (<0.001)
High back	0.88 (<0.001)	0.99 (<0.001)	0.93 (<0.001)	0.79 (<0.001)	0.98 (<0.001)
Back-row (zone 1)	0.89 (<0.001)	0.99 (<0.001)	0.86 (<0.001)	0.89 (<0.001)	0.98 (<0.001)

**Table 3:** Pearson’s coefficient (r) and Intraclass Correlation Coefficient (ICC) for the sets directed to the outside hitter performed in two sessions.

Joints Movement	Correlation and Reliability Coefficients (r, ICC)	Sets Directed to the Outside Hitter		
		High Ball	Super Ball	Pipe
Shoulder Flexion-Extension	r	0.9969 (<0.001)	0.9999 (<0.001)	0.9979 (<0.001)
	ICC (95% CI)	0.9963 [0.9950; 0.9972]	0.9903 [0.9896; 0.9928]	0.9976 [0.9969; 0.9981]
Shoulder Abduction-Adduction	r	0.9969 (<0.001)	0.9993 (<0.001)	0.9973 (<0.001)
	ICC (95% CI)	0.9929 [0.9904; 0.9947]	0.9891 [0.9853; 0.9919]	0.9940 [0.9923; 0.9953]
Shoulder Rotation	r	0.9941 (<0.001)	0.9995 (<0.001)	0.9955 (<0.001)
	ICC (95% CI)	0.9674 [0.9561; 0.9758]	0.9367 [0.9154; 0.9527]	0.9357 [0.9180; 0.9496]
Elbow Flexion-Extension	r	0.9895 (<0.001)	0.9908 (<0.001)	0.9965 (<0.001)
	ICC (95% CI)	0.9870 [0.9824; 0.9903]	0.9824 [0.9763; 0.9870]	0.9939 [0.9922; 0.9953]
Elbow Prono-Supination	r	0.9578 (<0.001)	0.9445 (<0.001)	0.9812 (0.0646)
	ICC (95% CI)	0.9309 [0.9077; 0.9484]	0.8378 [0.7870; 0.8773]	0.9556 [0.9432; 0.9653]
Wrist Flexion-Extension	r	0.9975 (<0.001)	0.9908 (<0.001)	0.9937 (<0.001)
	ICC (95% CI)	0.9941 [0.9920; 0.9956]	0.9865 [0.9818; 0.9900]	0.9414 [0.9252; 0.9541]

**Table 4:** Pearson’s coefficient (r) and Intraclass Correlation Coefficient (ICC) for the sets directed to the middle blocker performed in two sessions.

Joints Movement	Correlation and Reliability Coefficients (r, ICC)	Sets Directed to the Middle Blocker		
		Quick Set	Seven Set	Fast
Shoulder Flexion-Extension	r	0.9952 (<0.001)	0.9995 (<0.001)	0.9984 (<0.001)
	ICC (95% CI)	0.9942 [0.9921; 0.9958]	0.9984 [0.9977; 0.9988]	0.9974 [0.9965; 0.9981]
Shoulder Abduction-Adduction	r	0.9955 (<0.001)	0.9991 (<0.001)	0.9992 (<0.001)
	ICC (95% CI)	0.9858 [0.9804; 0.9897]	0.9913 [0.9879; 0.9937]	0.9945 [0.9926; 0.9959]
Shoulder Rotation	r	0.9958 (<0.001)	0.9996 (<0.001)	0.8686 (<0.001)
	ICC (95% CI)	0.9843 [0.9783; 0.9886]	0.9778 [0.9694; 0.9840]	0.8525 [0.8066; 0.8882]
Elbow Flexion-Extension	r	0.9806 (<0.001)	0.9938 (<0.001)	0.9649 (<0.001)
	ICC (95% CI)	0.9786 [0.9706; 0.9845]	0.9843 [0.9783; 0.9886]	0.9130 [0.8848; 0.9346]
Elbow Prono-Supination	r	0.9706 (<0.001)	0.9502 (<0.001)	0.8582 (<0.001)
	ICC (95% CI)	0.9354 [0.9119; 0.9528]	0.9162 [0.8857; 0.9389]	0.8412 [0.7921; 0.8795]
Wrist Flexion-Extension	r	0.9801 (<0.001)	0.9917 (<0.001)	0.9735 (<0.001)
	ICC (95% CI)	0.9298 [0.9044; 0.9487]	0.9515 [0.9333; 0.9648]	0.9486 [0.9315; 0.9615]

**Table 5:** Pearson’s coefficient (r) and Intraclass Correlation Coefficient (ICC) for the sets directed to the opposite hitter performed in two sessions.

Joints Movement	Correlation and Reliability Coefficients (r, ICC)	Sets Directed to the Opposite Hitter		
		High Back	Back-Row (Zone 1)	Two
Shoulder Flexion-Extension	r	0.9990 (<0.001)	0.9976 (<0.001)	0.9995 (<0.001)
	ICC (95% CI)	0.9754 [0.9671; 0.9816]	0.9962 [0.9948; 0.9972]	0.9989 [0.9984; 0.9992]
Shoulder Abduction-Adduction	r	0.9913 (<0.001)	0.9841 (<0.001)	0.9961 (<0.001)
	ICC (95% CI)	0.9804 [0.9738; 0.9854]	0.9573 [0.9429; 0.9682]	0.9855 [0.9800; 0.9895]
Shoulder Rotation	r	0.6724 (<0.001)	0.9188 (<0.001)	0.9522 (<0.001)
	ICC (95% CI)	0.5713 [0.4642; 0.6620]	0.5051 [0.3857; 0.6079]	0.6973 [0.6038; 0.7718]
Elbow Flexion-Extension	r	0.9959 (<0.001)	0.9901 (<0.001)	0.9902 (<0.001)
	ICC (95% CI)	0.9956 [0.9941; 0.9967]	0.9469 [0.9291; 0.9604]	0.9707 [0.9597; 0.9788]
Elbow Prono-Supination	r	0.8183 (<0.001)	0.7975 (<0.001)	0.8046 (<0.001)
	ICC (95% CI)	0.6099 [0.5094; 0.6940]	0.7653 [0.6959; 0.8205]	0.6991 [0.6061; 0.7732]
Wrist Flexion-Extension	r	0.9827 (<0.001)	0.9481 (<0.001)	0.9653 (<0.001)
	ICC (95% CI)	0.9426 [0.9237; 0.9569]	0.9137 [0.8853; 0.9353]	0.9492 [0.9304; 0.9631]

## Abbreviations

The following abbreviations are used in this manuscript:

EMG	Electromyographic
NMF	Non-negative matrix factorization
ICC	Intraclass Correlation Coefficient
r	Pearson’s coefficient

## References

[1] Fuchs P.X., Menzel H.J.K., Guidotti F., Bell J., von Duvillard S.P., Wagner H. (2019). Spike jump biomechanics in male versus female elite volleyball players. J. Sports Sci..

[2] Cabarkapa D.V., Cabarkapa D., Fry A.C., Whiting S.M., Downey G.G. (2022). Kinetic and Kinematic Characteristics of Setting Motions in Female Volleyball Players. Biomechanics.

[3] González-Silva J., Domínguez A.M., Fernández-Echeverría C., Rabaz F.C., Arroyo M.P.M. (2016). Analysis of Setting Efficacy in Young Male and Female Volleyball Players. J. Hum. Kinet..

[4] Denardi R.A., Romero Clavijo F.A., De Souza Santana T., Costa De Oliveira T.A., Corrêa U.C. (2024). The interpersonal coordination constraint on the volleyball setter’s decision-making on setting direction. J. Hum. Sport Exerc..

[5] Reeser J.C., Fleisig G.S., Bolt B., Ruan M. (2010). Upper Limb Biomechanics During the Volleyball Serve and Spike. Sports Health.

[6] Fatahi A., Sadeghi H., Yousefian Molla R., Ameli M. (2019). Selected Kinematic Characteristics Analysis of Knee and Ankle Joints During Block Jump Among Elite Junior Volleyball Players. Phys. Treat. Specif. Phys. Ther. J..

[7] Lin H.T., Huang Y.C., Li Y.Y., Chang J.H. (2021). The effect of rectus abdominis fatigue on lower limb jumping performance and landing load for volleyball players. Appl. Sci..

[8] Wagner H., Tilp M., Von Duvillard S.P.V., Mueller E. (2009). Kinematic analysis of volleyball spike jump. Int. J. Sports Med..

[9] Rokito A.S., Jobe F.W., Pink M.M., Perry J., Brault J. (1998). Electromyographic analysis of shoulder function during the volleyball serve and spike. J. Shoulder Elb. Surg. Board Trustees.

[10] Suda E.Y., Amorim C.F., de Camargo Neves Sacco I. (2009). Influence of ankle functional instability on the ankle electromyography during landing after volleyball blocking. J. Electromyogr. Kinesiol..

[11] Chen Y.-C., Huang C.-F. (2008). Kinematical analysis of female volleyball spike. ISBS Conference Proceedings Archive.

[12] Coleman S., Benham A.S., Northcott S.R. (1993). A three-dimensional cinematographical analysis of the volleyball spike. J. Sports Sci..

[13] Oliveira L.d.S., Moura T.B.M.A., Rodacki A.L.F., Tilp M., Okazaki V.H.A. (2020). A systematic review of volleyball spike kinematics: Implications for practice and research. Int. J. Sports Sci. Coach..

[14] Huang C., Hu L.-H. Kinematic analysis of volleyball jump topspin and float serve. Proceedings of the XXV ISBS Symposium, International Society of Biomechanics in Sports.

[15] Huang K.C., Hu U.H., Huang C., Sheu T.Y., Tsue C.M. (2002). Kinetic and Kinematic differences of two volleyball-spiking jump. ISBS Conference Proceedings Archive.

[16] Garcia S., Delattre N., Berton E., Divrechy G., Rao G. (2022). Comparison of landing kinematics and kinetics between experienced and novice volleyball players during block and spike jumps. BMC Sports Sci. Med. Rehabil..

[17] Ozawa Y., Uchiyama S., Ogawara K., Kanosue K., Yamada H. (2021). Biomechanical analysis of volleyball overhead pass. Sports Biomech..

[18] Ridgway M.E., Wilkerson J. (1986). A kinematic analysis of the front set and back set in volleyball. ISBS Conference Proceedings Archive.

[19] Adlou B., Wilburn C., Weimar W. (2025). Motion Capture Technologies for Athletic Performance Enhancement and Injury Risk Assessment: A Review for Multi-Sport Organizations. Sensors.

[20] Taborri J., Keogh J., Kos A., Santuz A., Umek A., Urbanczyk C., van der Kruk E., Rossi S. (2020). Sport biomechanics applications using inertial, force, and EMG sensors: A literature overview. Appl. Bionics Biomech..

[21] Alzahrani A., Ullah A. (2024). Advanced biomechanical analytics: Wearable technologies for precision health monitoring in sports performance. Digit. Health.

[22] Hermens H.J., Freriks B., Disselhorst-Klug C., Rau G. (2000). Development of recommendations for SEMG sensors and sensor placement procedures. J. Electromyogr. Kinesiol..

[23] Scano A., Dardari L., Molteni F., Giberti H., Tosatti L.M., D’Avella A. (2019). A comprehensive spatial mapping of muscle synergies in highly variable upper-limb movements of healthy subjects. Front. Physiol..

[24] Ghislieri M., Lanotte M., Knaflitz M., Rizzi L., Agostini V. (2023). Muscle synergies in Parkinson’s disease before and after the deep brain stimulation of the bilateral subthalamic nucleus. Sci. Rep..

[25] Pale U., Atzori M., Müller H., Scano A. (2020). Variability of muscle synergies in hand grasps: Analysis of intra- and inter-session data. Sensors.

[26] Rimini D., Agostini V., Knaflitz M. (2017). Intra-subject consistency during locomotion: Similarity in shared and subject-specific muscle synergies. Front. Hum. Neurosci..

[27] Brambilla C., Russo M., d’Avella A., Scano A. (2023). Phasic and tonic muscle synergies are different in number, structure and sparseness. Hum. Mov. Sci..

[28] Lee D.D., Sebastian Seung H. (1999). Learning the parts of objects by non negative matrix factorization. Nature.

[29] d’Avella A., Portone A., Fernandez L., Lacquaniti F. (2006). Control of fast-reaching movements by muscle synergy combinations. J. Neurosci..

[30] Weir J.P. (2005). Quantifying test-retest reliability using the intraclass correlation coefficient and the SEM. J. Strength Cond. Res..

[31] Lanzani V., Brambilla C., Scano A. (2024). Kinematic–Muscular Synergies Describe Human Locomotion with a Set of Functional Synergies. Biomimetics.

[32] Li J., Qiu F., Gan L., Chou L.S. (2024). Concurrent validity of inertial measurement units in range of motion measurements of upper extremity: A systematic review and meta-analysis. Wearable Technol..

[33] Roos R.E., Lambiase J., Riffitts M., Scholle L., Kulkarni S., Luck C.L., Parmanto D., Putraadinatha V., Yoga M.D., Lang S.N. (2025). The Reliability and Validity of an Instrumented Device for Tracking the Shoulder Range of Motion. Sensors.

[34] Pryhoda M., Newell K.M., Wilson C., Irwin G. (2022). Task Specific and General Patterns of Joint Motion Variability in Upright-and Hand-Standing Postures. Entropy.

[35] Robertson D.G.E. (2004). Research Methods in Biomechanics.

[36] Fleisig G.S., Escamilla R.F., Andrews J.R. (2011). Applied Biomechanics of Baseball Pitching.

[37] Tang L., Li F., Cao S., Zhang X., Chen X. Muscle Synergy Analysis for Similar Upper Limb Motor Tasks. Proceedings of the 2014 36th Annual International Conference of the IEEE Engineering in Medicine and Biology Society.

[38] Scano A., Lanzani V., Brambilla C. (2024). How Recent Findings in Electromyographic Analysis and Synergistic Control Can Impact on New Directions for Muscle Synergy Assessment in Sports. Appl. Sci..

[39] Safavynia S., Torres-Oviedo G., Ting L. (2011). Muscle synergies: Implications for clinical evaluation and rehabilitation of movement. Top. Spinal Cord Inj. Rehabil..

[40] Matsuura Y., Matsunaga N., Akuzawa H., Kojima T., Oshikawa T., Iizuka S., Okuno K., Kaneoka K. (2022). Difference in muscle synergies of the butterfly technique with and without swimmer’s shoulder. Sci. Rep..

[41] Thomas S.J., Castillo G.C., Topley M., Paul R.W. (2023). The Effects of Fatigue on Muscle Synergies in the Shoulders of Baseball Players. Sports Health.

